# Epidemiology of Uterine Myomas: A Review 

**DOI:** 10.22074/ijfs.2015.4599

**Published:** 2015-12-23

**Authors:** Radmila Sparic, Ljiljana Mirkovic, Antonio Malvasi, Andrea Tinelli

**Affiliations:** 1 Clinic for Gynecology and Obstetrics, Clinical Center of Serbia, Belgrade, Serbia; 2 Faculty of Medicine, University of Belgrade, Belgrade, Serbia; 3 Department of Obstetrics and Gynecology, Santa Maria Hospital, Bari, Italy; 4 International Translational Medicine and Biomodelling Research Group Department of Applied Mathematics, Moscow Institute of Physics and Technology (State University), Moscow Region, Russia; 5 Division of Experimental Endoscopic Surgery, Imaging, Technology and Minimally Invasive Therapy, Department of Obstetrics and Gynecology Vito Fazzi Hospital, Lecce, Italy

**Keywords:** Uterine Myoma, Fibroid, Leiomyoma

## Abstract

Myomas are the most common benign tumors of the genital organs in women of childbearing age, causing significant morbidity and impairing their quality of life. In our investigation, we have reviewed the epidemiological data related to the development of myomas in order to homogenize the current data. Therefore, a MEDLINE and PubMed search, for the years 1990-2013, was conducted using a combination of keywords, such as "myoma," "leiomyoma," "fibroids," "myomectomy," "lifestyle," "cigarette," "alcohol," "vitamins," "diet," and "hysterectomy". Randomized controlled studies were selected based upon the authors’ estimation. Peer-reviewed articles examining myomas were sorted by their relevance and included in this research. Additional articles were also identified from the references of the retrieved papers and included according to authors’ estimation.

Many epidemiologic factors are linked to the development of myomas; however, many are not yet fully understood. These factors include age, race, heritage, reproductive factors, sex hormones, obesity, lifestyle (diet, caffeine and alcohol consumption, smoking, physical activity and stress), environmental and other influences, such as hypertension and infection. Some of the epidemiological data is conflicting. Thus, more research is needed to understand all the risk factors that contribute to myoma formation and how they exactly influence their onset and growth.

## Introduction

Myomas are the most common benign neoplasm of the reproductive organs in women of reproductive age. They could have a negative impact on the reproductive system and can be single, but are more often multiple, causing significant morbidity, and deterioration of quality of life (1,2). According to relevant literature, 40-60% of all the hysterectomies performed are because of the presence of myomas. Myomas are the most common indication for hysterectomy in the USA and Australia (3,4). 

**Fig.1:**
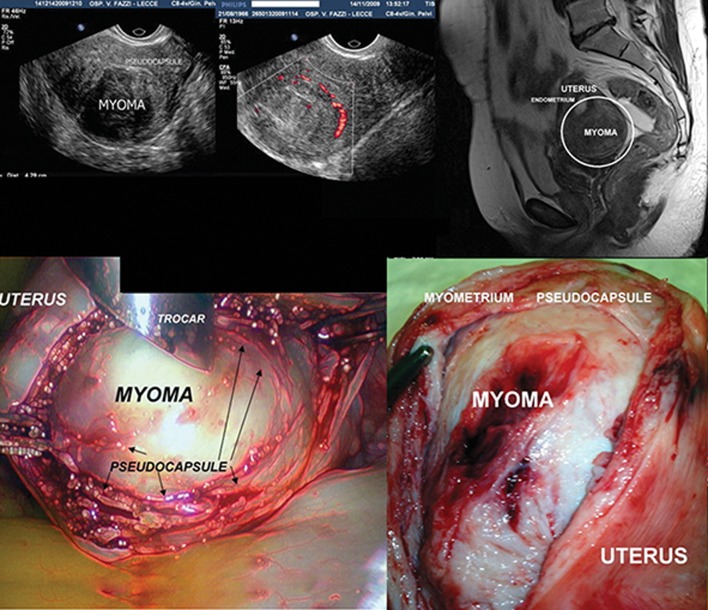
A composed image in clockwise fashion showing: A. Transvaginal transversal scan showing a posterior corporal myoma, B. An eco Doppler transvaginal scan detecting the myoma pseudocapsule as a "ring of fire", C. A T2 pelvic MRI showing a posterior corporal myoma enhanced by a white ring, D. Laparoscopic image showing the myoma enucleation surrounded by pseudocapsule. The arrows indicate the myoma pseudocapsule, as a fibrovascular connective network surrounding myoma and E. A laparotomic image showing a large uterine myoma surrounded by pseudocapsule during enucleating from myometrium.

Matthew Baille was the first to describe myomas in 1793. Myomas consist mainly of smooth muscle cells and contain different amounts of fibrous tissue (5). During its growth, a myoma compresses the surrounding structures (the myometrium and connective tissue), causing the progressive formation of a sort of pseudocapsule, rich in collagen fibers, neurofibers and blood vessels (Fig .1). Occasionally, the continuous surface of the pseudocapsule is interrupted by bridges of collagen fibers and vessels that anchor the myoma to the myometrium. This causes the formation of a clear cleavage plane between myoma and the pseudocapsule, and between the pseudocapsule and the surrounding myometrium. This pseudocapsule causes a displacement action (which is not destructive) on the myometrium; however, the integrity and contractility of uterine structure is maintained (6, 7).

Literature data has shown that between 5.4 to 77% of women have myomas, depending on either the study population or the diagnostic techniques applied (8). Studies conducted using the ultrasound have confirmed that myoma prevalence is lower in Europe than in the United States, and this is probably due to racial differences (9, 10). Myomas are detected in 70% of uteri after hysterectomy, where multiple myomas are present in more than 80% of cases (11). Myoma prevalence was largely underestimated in previous epidemiological studies that focused mostly on symptomatic women (5, 10-12). By using more advanced non-invasive imaging techniques, such as 3D-4D ultrasonography (US) screening on the general population, epidemiological studies have become more accurate over the past two decades (1, 10) . Thus, Laughlin et al. (13), reported a lower myoma prevalence of 10.7% in women screened in the first trimester of pregnancy.

The data on epidemiologic factors associated with myoma risk are either well defined or not yet fully understood (10). Those factors include age, race, body mass index (BMI), heritage, reproductive factors, sex hormones, obesity, lifestyle (diet, caffeine and alcohol consumption, smoking, physical activity and stress), environmental and other impacts like hypertension and infection (1, 10). The reported impacts of these factors in literature are conflicting (10, 12, 14). This could be attributed to bias in patient selection, given that some of the studies are based on surgical or symptomatic cases, while others on the incidental diagnosis of myomas (10).

## Discussion

In this article, we have investigated the available epidemiological data regarding myoma development. For this purpose a MEDLINE and PubMed search, for the years 1990-2013, was conducted using a combination of keywords, such as "myoma," "leiomyoma," "fibromyoma", "leiomyofibroma", "fibroleiomyoma", "fibroid," "myomectomy,", "lifestyle," "cigarette," "alcohol," "vitamins," "diet," and "hysterectomy". Randomized controlled studies were used when available; otherwise, literature that was the most relevant to the topic was used based on the authors’ estimation. Peer-reviewed articles regarding myomas, fibroids and leiomyomas were included in this paper. Additional articles were identified from the references of relevant papers. The terms "leiomyomas", "fibroids", "fibromyomas", "leiomyofibromas" and "fibroleiomyomas" can also be found in the literature describing myomas (15). In this paper, we have used the term myoma. The aim of this review is to provide information about epidemiological data regarding myoma development and make it more homogenous.

### Age

During the reproductive years, the risk of myoma development increases with age (10). Myomas do not occur before puberty and their frequency decreases with menopause (16, 17). Myomas are diagnosed in 20-25% of women of reproductive age, and 30-40% of women older than 40 years (1, 4, 5, 18). Women with an earlier age of menarche have a higher risk for uterine myoma development (5, 10). It is to be expected that late-onset menopause increases risk of myoma occurrence due to longer exposure to gonadal steroids. However, the epidemiological data on this is still insufficient (10). The clinical incidence of myomas, in terms of a symptomatic disease requiring treatment, is the most frequent in perimenopause, whereas after menopause it rapidly decreases (19).

### Race

Myomas are the most common in women of the black race, and the rarest in women of the Asian race (5). The data regarding racial differences other than in Caucasian and African American women are limited (10, 20). Laughlin et al. (13) determined the following prevalence: 18% in black women, 8% in white women, 10% in Hispanic women and 13% in the "others" group, consisting largely of Asian women. Black women are usually diagnosed at a younger age, with myomas that are often multiple, larger and accompanied by more severe symptoms than in other ethnic groups (10, 12, 16). Thus, black women are subjected to hysterectomies and myomectomies at an earlier age than white women (5). Myoma regression after pregnancy occurs more often in white women than in black (1). In addition, the myoma growth rate is slower as age progresses in white women than in black women (20).

The exact reasons for racial variations in the occurrence of myomas are mostly unknown. In literature, as the possible cause given for this phenomenon are the racial differences in the biosynthesis and/or metabolism of estrogens. Differences in the expression and/or function of receptors for steroid hormones among races can be considered as another possible cause of ethnic differences in myoma incidence (16). Aberrant expression of micro-RNA is another possible molecular mechanism involved in the development of myomas (16, 20). Micro-RNAs are a class of small noncoding RNAs important in the regulation of cell proliferation, differentiation and death, and their expression shows significant differences in various ethnic groups (16). Other causes analyzed in literature include heritage, lifestyle, dietary habits, and stress. However, these factors, can only somewhat explain the racial differences in myoma occurrence and their growth rates (10, 14, 20-25). By examining the data on why various races and ethnic groups have an increased risk of myoma development, new facts may be discovered regarding the etiology, formation and growth mechanisms of myomas, which could lead to new strategies for their assessment and treatment (20).

### Genetics

Genetic factors can play a significant role in myoma development (5, 26). The growth of multiple myomas in the same uterus implies that heritage plays an important role in myoma development, causing some women to be more predisposed than others. The existence of the so-called "myoma families" (19, 26) described in literature proves a familial predisposition to myoma formation. Uimari et al. (26), in Finland, observed that in cases of familial myomas women were diagnosed at an earlier age and more commonly with multiple myomas, so they tended to undergo hysterectomies at a younger age as well. Studies on twins have revealed a greater risk of myoma formation in monozygotic than in dizygotic twins (5, 27). The high myoma recurrence rate following myomectomy indicates that women with myomas have an inherited gene or some other genetic predisposition to myoma development. Cytogenetic analysis of the myoma cells proved the existence of tumor-specific chromosomal abnormalities in approximately 40% of the tested samples (10). Cytogenetic analysis of multiple myomas from the same uterus may show different chromosomal changes, which can mean that each myoma develops independently (5) and that certain regions of the genome may be involved in the pathogenesis of the myomas.

It is known that somatic mutations involving the gene encoding the mediator complex subunit 12 (MED12) and the gene encoding the high-mobility group AT-hook 2 (HMGA2) are associated to myoma (28). Mäkinen et al. (29) found that approximately 70% of myomas had heterozygous somatic mutations that affect MED12, transcriptional regulator complex subunit 12, a gene located on the X chromosome. The authors demonstrated that all mutations resided in exon 2 (codon 44), suggesting that the aberrant function of this region of MED12 contributes to tumorigenesis.

Since genetic analyses have supported the idea of a genetic component in myoma predisposition, Eggert et al. (30) genotyped and analyzed a genome-wide single nucleotide polymorphisms (SNP) linkage panel in 261 white myomas-affected sister-pair families from the Finding Genes for Fibroids study. All women were from two cohorts. The first was the Women’s Genome Health Study (WGHS), a prospective cohort of female North American health-care professionals representing Women’s Health Study (WHS) participants who provided a blood sample at baseline and consent for blood-based analyses. The second cohort was from the Queensland Institute of Medical Research (QIMR). Two significant linkage regions were detected in 10p11 and 3p21, and five additional linkage regions were identified in 2q37, 5p13, 11p15, 12q14, and 17q25. They performed genome-wide association studies (GWASs) in two independent cohorts of white women, conducting a meta-analysis. One SNP (rs4247357) was identified as having genome-wide significance. Authors showed elevated (3-fold) spans fatty acid synthase (FAS) levels in myoma-affected tissue compared to matched myometrial tissue by tissue microarray immunohistochemistry. FAS represents the initial myoma risk allele identified in white women by a genomewide, unbiased approach and opens a path to management and potential therapeutic intervention. Heritage is also suggested to be a possible reason of the racial differences (10). From 1997 to 2009, Wise et al. (14) carried out a national prospective cohort study, in which 2,453 myomas from an admixture- based genome-wide were scanned. This was conducted in order to investigate the presence of risk alleles for myomas that are very different in frequency between African and European Americans. This investigation was the first genome-wide association scan for myomas in African Americans and the first admixture mapping study of myomas in any population. In the results, the mean percentage of European ancestry was significantly lower among cases than among controls, with a stronger association in younger cases, less than 35 years old at diagnosis. Furthermore, the authors found only suggestive evidence for an association with European ancestry at specific loci (chromosomes 2, 4, and 10), with stronger results among younger and surgical cases for chromosome 2 only. This feature implied that a genetic variation for myomas differs in populations with and without African ancestry. The admixture findings further indicated that no single highly differentiated locus is responsible for the ethnic disparity in myomas, raising the possibility that multiple variants jointly contribute to the higher incidence of myomas in African Americans. Nevertheless, authors failed to replicate results from a recent GWAS in Japanese women by Cha et al. (31). In this investigation, authors reported a case-control GWAS that aimed to identify common genetic variants associated with uterine myomas. In this GWAS, the authors examined 1,612 individuals who were clinically diagnosed to have myomas at affiliated hospitals of the BioBank Japan Project and 1,428 female controls without a history of uterine myomas. They analyzed 457,044 SNPs in all patients. Three loci on chromosomes 10q24.33, 22q13.1 and 11p15.5 revealed genomewide significant associations with myomas. The SNPs showing the most significant association in a combination analysis at each of these loci were rs7913069, rs12484776 and rs2280543, respectively. Moreover, to assess whether these loci could be associated with clinically symptomatic myomas or with related phenotypes of the disease, authors performed subgroup analyses, founding that each marker SNP consistently showed a strong association with myoma formation regardless of presence or absence of hypermenorrhea or dysmenorrhea. These results indicated that these SNPs were associated with the development of myomas but not with the progression of disease.

After the Cho’s study, Edwards et al. (32) tested these SNPs for association with myomas in US cohorts. At patients’ enrollment, a transvaginal ultrasound was conducted to assess embryonic development and to systematically examine the uterus for presence of myomas. Patients were from a community-based pregnancy cohort that was carried out between 2001 and 2012, the Right from the Start (RFTS) cohort and the BioVU DNA repository. The authors tested 65 candidates and haplotypetagged SNPs for association with myoma presence, and combined associated results from both cohorts using meta-analysis. Authors analyzed 1,086 European American cases and 1,549 controls. They observed strong evidence of association across several markers with transport 1 homolog (BET1L) and trinucleotide repeat containing 6B (TNRC6B), including two of the previously associated GWAS index SNPs. Metaanalyses combining evidence from RFTS, BioVU, and prior GWAS showed little heterogeneity in effect sizes studies, with meta-p values between 7.45×108 and 3.89×109, which were stronger than prior GWAS and supported associations observed for all previously identified loci. This data suggests that common variants increase risk for myomas in both European American and Japanese populations, even if further research is needed to assess the role of these genes across other racial groups.

### Reproductive factors

The inverse association between myoma risk and parity is well known (5, 10, 12, 33) and an increasing number of term pregnancies decreases myoma risk. Both hormonal and non-hormonal mechanisms may also explain this association. Parity means decreased menstrual cycling and term pregnancies cause changes in ovarian hormones, growth factors and estrogen receptor levels, and changes in the uterine tissue (12). Thus, myomas are more common in nulliparous women, although excess weight and obesity seem to lessen the inverse association with parity (10, 12). Myoma development risk is reduced with the older age of the woman in last term pregnancy. Results from Nurses’ Health Study II have documented that myoma risk is reduced with the older age of the woman at the first birth and the last birth, and the more recent with the last birth (33). The study of Wise et al. (12), in African American women showed that time since the most recent birth is positively related to myoma risk among parous women. This observation can be explained by non-hormonal causes, such as postpartum tissue changes during uterine involution process (10). Increased risk for myomas is associated with early menarche and older age of the first term of pregnancy (5). The cause of this is thought to be increased exposure to menstrual cycles during a nulliparous woman’s lifetime, uninterrupted by pregnancy and lactation. This is also a plausible explanation for early menarche. Pregnancies that did not reach full term seem to have no influence on myoma formation risk (5, 12). Among multiparous women, the inverse association between myoma risk and exclusive breastfeeding throughout life was demonstrated by Terry et al. (33). This can be explained by the fact that lactation suppresses ovarian hormones. On the contrary, Wise et al. (12) did not find either lactation or its duration to be a protective factor in myoma development in African American women. This may be explained by the fact that breastfeeding happens only during a short period of a woman’s lifetime to have any significant impact on myoma development. It is not clear why pregnancy causes a reduction in myoma risk, but it can be that the postpartum physiological involution of the uterus eliminates myomas or reduces their size after delivery (10, 34, 35). This is confirmed by the recently published data (10).

### Endogenous hormones

Myomas occur only during the reproductive period, which proves their dependence on ovarian steroids (36). The fact that estrogen and progesterone are significant in myoma onset and growth is evident in both clinical and experimental studies (10, 12). How they exactly influence myoma formation and growth is not yet fully understood (37). Early menarche increases the risk of myomas, due to longer exposure to circulating ovarian steroids over a lifetime. Estrogen is believed to promote the growth of myomas (12). Recent researches have indicated that progesterone may also be important for the growth of myomas, because it acts synergistically with estrogen to stimulate myoma (10). For such reasons, selective progesterone receptor modulators (SPRMs), such as asoprisinil, ulipristal and telapristone have been researched as potential therapeutic drugs for uterine myomas (38). Ulipristal acetate (UPA) has demonstrated promising results for becoming a suitable therapeutic drug for uterine myomas. Results of international randomized controlled trials (PEARL I and PEARL II) showed that UPA decreased the size of the myomas and reduced bleeding, while increasing the red blood cell count after three months’ use of 5 mg/day (38, 39). Thus, UPA has been registered in some countries for the preoperative treatment of myomas for a period up to three months.

Myoma risk correlates with increased luteinizing hormone (LH) levels. Literature data indicate a positive association between polycystic ovary syndrome (PCOS) and myomas (5, 10, 40). A 65% higher incidence of myomas in women with PCOS compared with those without it, even after adjustment for potential confounding factors, was determined in the Black WHS (BWHS). The drawback of this study documenting the positive association between the PCOS and myomas in African American women is that the PCOS was self-reported. The LH hypothesis is also supported by the finding that the effect of PCOS is stronger among lean than in obese women. The explanations for this association are insulin resistance and elevated levels of insulin-like growth factor I (IGF-I), and hyperandrogenism (40). Still, Wise et al. (40) failed to determine that diabetes modified the association between myomas and PCOS.

### Exogenous hormone use

The relationship between oral contraceptives and myomas has been widely researched (10, 12). Epidemiological data on the relationship between the use of oral contraceptives and myomas is inconsistent (17, 41). Oral contraceptive use may enhance diagnosis due to detection bias. Published studies show either a reduced or an absence of risk between the use of combined oral contraceptives and the occurrence of myomas (41). Thereby, according to Wise et al. (12), there is no link between the use of oral contraceptives and the risk of myoma in African American women. In this study, myoma risk was influenced by neither the ingredients of oral contraceptive nor its hormonal strength, not by duration or recency of use. A slightly higher risk is related to the age of first oral contraceptive use. This study shows a decreased risk of myomas in current users of progestin-only injectables. The reason for this is downregulation of the estrogen receptors in myomas caused by progestin (12).

The effects of IUDs with the levonorgestrel and risk of myoma development is still unknown (41, 42).

In postmenopausal women receiving hormone replacement therapy, both in women receiving estrogens only and in those receiving combined therapy, there is an increased occurrence of myoma growth (10).

Another factor that could also contribute to myoma risk is exogenous hormones in food. They could be in the form of the so-called phytoestrogens, as well as of those of artificial origin (24).

Diethylstilbestrol (DES) exposure studies are influenced by reporting bias; therefore, their findings are conflicting (10). Further research in this field is needed by means of well-designed studies. This is necessary as laboratory data indicate a positive association, while clinical reports documented both positive association and absence of any association (10).

### Obesity

The relationship between obesity and myoma development has shown to be inconsistent in literature (5, 40). Some epidemiological studies have found the increased risk of myoma development to be associated with obesity and diabetes mellitus (5, 10, 17, 40, 43). The common factor contributing to this association is insulin resistance, which is believed to be responsible for myoma risk developing in obese women, together with elevated IGF-I and androgen levels (5, 44).

A significantly higher BMI in women with myomas was documented in the Finnish twin cohort study (27). This can be explained by the presence of increased levels of circulating estrogens, caused by the aromatization of androgens by peripheral fatty tissues in obese women (44). However, most of the circulating estrogens originate from ovaries in premenopausal women, which questions this theory (10). Certainly, what can be considered as a contributing factor in high myoma risk in these women is the decreased hepatic production of sex hormone binding globulin (SHBG), resulting in increased bioavailability of estrogens and androgens (5, 10, 44). He et al. (45) also found an increased risk of myomas in premenopausal Asian women with a high BMI. However, Chiaffarino et al. (46), in Italy, did not find any association between BMI and the risk of myomas.

### 

In the US, obesity is prevalent among black rather than among white women. Thus, obesity is believed to be one of the reasons for the racial differences in the risk of myoma development. The results from the BWHS revealed a complex non-linear, but inverse J-shaped pattern between BMI and myoma risk (25). This connection appears to depend on parity, extent of obesity, and detection bias. There is also a positive association between myoma risk and weight gain during adulthood (10). In both white and black women, the association between the BMI of overweight women and myoma risk was found to be stronger in surgically confirmed cases (10, 25). In the US, both in white and black women, an absence of association was found between height and myomas (25).

### Lifestyle

Lifestyle factors, such as diet, caffeine and alcohol consumption, smoking, physical activity, and stress have a potential effect on the formation of myomas and their growth (45). For easier reporting, we have divided the results of our research into subheadings.

### Diet

The study results investigating the impact of diet on the occurrence of myomas are inconclusive, due to selection biases and the presence of confounding factors (10). Differences in diet could partly explain the racial differences in the prevalence of myomas. Therefore, in African American women myomas are more frequent, and they consume less fruit, vegetables, vitamin and mineral supplements (21, 22). Several dietary factors have been shown to contribute to the development of symptomatic myomas (45). Myoma formation risk is slightly higher in women consuming food with a higher glycemic index. Vitamins A and D are potential protective factors. Soy food was claimed to have an inverse relationship with myomas, but researches in this area have failed to find this association (22, 45). Furthermore, they have also failed to prove reduced myoma risk in populations with a high soy intake (10).

### Meat

Current data demonstrate a positive link between a diet rich in red meat and myoma incidence (17). Chiaffarino et al. (46) conducted a case-control study of surgically confirmed cases in Italy, which demonstrated that women with myomas had a higher intake of beef, other red meat and ham and a lower intake of green vegetables, fruit and fish. Data obtained in this study are difficult to interpret due to several biases. Recently, Wise et al. (23) published the results on the relation of dietary fat intake and myoma risk in African American women, confirming an increased risk associated with the intake of long-chain omega-3 fatty acids, specifically marine fatty acids (MFA). Dark-meat fish was the main source of MFA in this study. Nevertheless, a dose-response relation for dark meat fish was not established. The overall risk of myoma has not been associated with total fat and fat subtypes intake in this study.

### Fruit and vegetables

Wise et al. (21) validated that a diet rich in fruit and vegetables reduced the risk of myomas, especially one rich in fruits. Women who consumed a high amount of citrus fruits had a much smaller risk of myoma. The inverse association between myomas and vegetable and fruit intakes was also recorded by He et al. (45) in a study conducted in Beijing. The protective effect of a high intake of green vegetables and fruit was reported by Chiaffarino et al. (46) in Italy. They suggested that a higher intake of vegetables, fruit and fish indicates healthier dietary and lifestyle habits. The limitation of this study is the absence of total energy intake data, as information was collected only on frequency of vegetable intake, and during interviews with patients after they had been diagnosed with myoma (46).

### Dairy

In a case-controlled study, Chiaffarino et al. (46) determined a null association between milk and butter consumption and myoma risk. In fact, investigations from the BWHS showed an inverse association of calcium, phosphorus and calcium-to-phosphorus ratio with myoma risk (10). The data from BWHS documented an inverse association of both low fat and high-fat milk with myoma risk. Thus, Wise et al. (22) concluded that racial differences in myoma incidence could be a result of differences in dairy intake. A subsequent paper by the same authors (24) noted that this relation could not be attributed to African ancestry.

### Micronutrients

There is limited data about the effects of micronutrients on myoma formation and development, thereby the exact mechanisms involved in this association are not yet fully understood (47).

Dietary intake of vitamins C or E and folate were not found to be associated with myoma formation risk (21). Furthermore, the intake of vitamin B6, vitamin B12, folate and vitamin E were also not proven to have any association with myoma formation. Martin et al. (47) did not find vitamins A and C to reduce the risk of myoma formation either.

### Vitamin D

Hypovitaminosis D, both in black and white women, is postulated as a potential risk factor in the myoma formation (48). Vitamin D is a fat-soluble steroid generated in the skin from a precursor molecule after sunlight exposure, or assumed in dietary foods (sometimes artificially enriched in vitamin D). Laboratory and animal evidence demonstrate that 1,25-dihydroxyvitamin D3 inhibits myoma growth and induces apoptosis (49). Recent research by Baird et al. (48) concluded that women with sufficient vitamin D have a reduced risk of myoma in comparison with women with vitamin D deficiency, and this was shown to be similar for both black and white women. African American women, who have a higher incidence of vitamin D deficiency, also have a higher frequency of myoma. Insufficient and inconclusive data in literature regarding this topic requires further research in this field.

### Vitamin A

The data analyzing the relation between myoma and vitamin A are rare. A positive association between vitamin A and myoma formation was determined by Martin et al. (47). They have demonstrated a dose-response relationship between serum levels of vitamin A and myoma development odds. The limitations of their study are a self-reported myoma status and potential changes to the participants’ dietary habits following myoma diagnosis.

Wise et al. (21), demonstrated an inverse association of dietary vitamin A intake and myoma risk in black women. However, this association was present only when the intake of vitamin A is derived from animal products, while it was absent when the total vitamin A intake was from other sources. Thus, they concluded that the risk reduction was caused from other ingredients, rather than from the vitamin A in the food.

### Carotenoids

Carotenoids are fat-soluble pigments found in many fruits and vegetables (50). They are powerful antioxidants, and some have pro-vitamin A activity, of which lycopene has the strongest antioxidant properties without any vitamin A activity. Animal studies have demonstrated that diets supplemented with lycopene reduce the number and size of myomas in a dose dependent manner (51). Literature data analyzing lycopene effect on myoma growth in humans is scarce. According to Terry et al. (50), the risk of myoma diagnosis is not associated with dietary carotenoids. The absence of association between myoma risk and carotenoid intake was also documented by Wise et al. (21).

### Bioflavonoids

Myoma frequency is lower in Asian women because they consume more soy food products, which are rich in isoflavones, than other races (52). Although phytoestrogens found in soy foods were believed to reduce myoma risk, He et al. (45) did not find any relation between soy products and myoma risk in Asian women. No relation between soy intake and myomas was also confirmed in a study conducted by Nagata et al. (52) in Japan. Data consistent with those two studies were provided by Atkinson et al. (53), who did not find any connection between isoflavone urinary excretion and myomas in a population with a low intake of soy foods.

The results of experimental studies on myoma cell lines demonstrated that flavonoids from Scutellaria barbata D. Don induce apoptosis and inhibit cell proliferation (54). This makes flavonoids from the Asian herb possible substances for developing anti-myoma medications in the future.

Green tea extract has shown to inhibit proliferation and induce apoptosis on myoma cells in animal studies (55). Gallactocatehin gallate (EGCG), an extract (catehin) of green tea, has been proven to inhibit cell proliferation on cultured human leiomyoma cells in a dose-and time-dependent manner (56). Thus, EGCG needs to be further researched as a potential drug for myoma treatment.

### Caffeine and alcohol

Literature data indicate that both caffeine and alcohol can change endogenous hormone levels (10, 57). Alcohol consumption has been proven to increase the risk of myoma (52, 57). A positive association between alcohol consumption and risk of myomas was confirmed in Japanese women (52). In the BWHS, Wise et al. (57) found the association to be stronger in beer drinkers, rather than in wine drinkers. Chiaffarino et al. (46) in Italy did not notice any association between myoma risk and the intake of coffee, tea or total alcohol consumption. The reason for the absence of such an association could be the fact that wine accounted for more than 90% of the alcohol consumed in this study. In African American women, Wise et al. (57) did not find any association between coffee and caffeine consumption and myoma risk. More research is needed in order to determine the link between myoma risk and caffeine and alcohol consumption, given that these risk factors may be modifiable.

### Smoking

The studies showing the relation between cigarette smoke and myoma risk are overall inconsistent (57). In earlier epidemiological studies, current or former smokers had a 20-50% (10) decreased risk of myomas compared to non-smokers, which suggested a protective effect of smoking on myoma formation (5, 10, 17, 43, 58). More recent and better-designed studies have not documented such a relationship (10). Dragomir et al. (59) conducted a research on both black and white American women, which revealed a positive association between current smoking and diffuse myomas. However, this association was absent in cases with either submucosal or intramural/subserosal myomas. How smoking influences myoma formation is not entirely clear and further research is necessary (5, 10).

### Physical activity

There have been few studies investigating the effect of physical activity on the risk of myoma development. Despite this, a reduced risk of myoma formation was determined in women who take physical exercise and have a normal body weight (17). In women who take regular physical exercise, the risk of myoma is lower compared to women who do not exercise (10). Baird et al. (60) also demonstrated an inverse association in both black and white women regarding current physical activity and myoma development, where there is a stronger relation to myoma onset than to myoma growth. In Asian women, He et al. (45) found a marginal association between myomas and weekly physical activity non-related to women’s occupation. Women with moderate intensity of physical activity related to work had significantly lower myoma development risk. Given that this is a modifiable risk factor, more research is necessary to assess the effects of physical activity on myoma biology.

### Stress

Stress can also be a potential risk factor in myoma formation (61, 62). However, data is lacking on this topic. Stress could lead to myoma formation causing the increase of estrogen and progesterone levels, due to the effect on the hypothalamopituitary- adrenal gland axis activation and release of cortisol, a stress hormone (62). For example, black women who have experienced stress resulting from racial discrimination are more likely to have myomas. The potential reasons for this association are heavy alcohol consumption, poor diet, and obesity (61). The association between major life stress and myomas was also analyzed by Vines et al., who explored both the number of major life events experienced and the stress intensity associated with those events in relation to myoma presence. A positive association with myomas among black women in the high stress intensity group was shown by the cited authors (62). In the Asian population, no association between myomas and stress, depression and feelings of anxiety was documented (45). 

### Environmental factors

Myomas are believed to develop under the influence of environmental factors, such as irradiation. Studies have shown a significantly higher myoma incidence in women who survived the atomic explosion, the incidence being dependent on the dose of irradiation (63). 

### Other factors

#### Hypertension and diabetes

Several epidemiological studies found the increased risk of myomas in women with diabetes mellitus and arterial hypertension (5,10,17,37,43,44). While experimental studies demonstrated stimulation by IGF-I of proliferation of myoma cells in the culture, clinical studies did not prove the association between myoma risk and plasma levels of IGF-I (10,37). No association between circulating insulin levels and the presence of myomas was determined in both black and white women according to Baird et al. (37). Furthermore, elevated insulin was shown to be protective for large myomas, particularly among the black population. An inverse association between diabetes and myoma risk was confirmed in different studies. Wise and Laughlin-Tommaso (10) documented it in black women and Baird et al. (37) in both black and white women. Myoma development is thought to be inhibited by systemic vascular dysfunction in women with diabetes. 

The coexistence of uterine myomas with hypertension was noted since the 1930s (64). Thus, hypertension has been considered as a risk factor for myoma development (20). Hypertension in women with myomas is usually chronic and requires treatment with antihypertensive drugs (64). In the study conducted by Boynton-Jarrett et al. (65) on women in the Nurses’ Health Study II cohort, an association was determined between higher diastolic pressure and myoma risk regardless of antihypertensive drug use. According to the results of this study, the duration of hypertension also increased myoma formation risk. To explain such an association, the authors suggested that hypertension may have caused cytokine release or injury to the smooth muscle of the uterus (44,65).The results of those studies may be questioned in terms of possible screening and intervention biases, as they investigated symptomatic or surgery -confirmed cases (10,64,65). To further evaluate this association, it is necessary to conduct more research (10). 

#### Infection and uterine injury

Infection or irritation causing uterine injury and followed by a disordered healing process was assumed as a possible reason for myoma formation in the first half of the twentieth century. It was suggested that uterine injury could induce changes in various growth factors causing myoma formation onset (10). 

One study suggested that the use of perineal talc acting as a possible uterine irritant is associated with myoma formation. This case-controlled study documented a positive association both for frequency and duration of use (64). Another casecontrolled study from Brazil showed an association between the Chagas disease and myomas in multiparous white women subjected to surgery either for myoma presence or for uterine prolapse (66). Faerstein et al. (64) showed a dose-response relation between ultrasound or surgically confirmed diagnosis of myomas and a number of physician diagnosed episodes of pelvic inflammatory disease (PID). Chlamydia infection was associated with a non-significant increase of myoma diagnosis in this study. This case-controlled study failed to establish an association between myoma and genital herpes or warts. It is necessary to conduct more studies in order to determine the relation between abnormal wound healing and myoma formation. 

## Conclusion

Clearly more research is necessary to determine the risk factors associated with myoma onset and growth considering that they cause significant morbidity and impair the quality of life. Clear insight into myoma epidemiology has not yet been achieved, and future research into modifiable risk factors may shed light on myoma prevention and provide new approaches to non-surgical myoma treatment. 
